# Medical Journal Changes

**Published:** 1858-09

**Authors:** 


### MEDICAL JOURNAL CHANGES.

*The Medical and Surgical Reporter*, of Philadelphia, has been changed from the form of a monthly to that of a weekly sheet. It remains under the efficient editorial management of Drs. Butler and Atkinson.

*The Nashville Medical Record* has been commenced as the successor of the *Memphis Medical Reporter* and the *Southern Journal of Medical and Physical Sciences.* It is edited by Drs. Wright and Curry, professors in the new Shelby Medical College, and issued monthly.

